# Detection and infectivity of SARS-CoV-2 in exhumated corpses

**DOI:** 10.1007/s00414-021-02670-4

**Published:** 2021-07-24

**Authors:** S. Plenzig, F. Holz, D. Bojkova, M. Kettner, J. Cinatl, M. A. Verhoff, C. G. Birngruber, S. Ciesek, H. F. Rabenau

**Affiliations:** 1 Institute of Legal Medicine, Goethe University, University Hospital Frankfurt, Kennedyallee 104, 60596 Frankfurt am Main, Germany; 2 Institute of Medical Virology, Goethe University, University Hospital Frankfurt, Paul-Ehrlich-Straße 40, 60596 Frankfurt am Main, Germany; 3 German Centre for Infection Research, External Partner Site, 60323 Frankfurt am Main, Germany; 4 Branch Translational Medicine and Pharmacology, Fraunhofer Institute for Molecular Biology and Applied Ecology (IME), 60596 Frankfurt am Main, Germany

**Keywords:** COVID-19, Autopsy, RT-PCR-detection, Cell culture

## Abstract

Postmortem detection of severe acute respiratory syndrome coronavirus type 2 (SARS-CoV-2) after the exhumation of a corpse can become important, e.g. in the case of subsequent medical malpractice allegations. To date, data on possible detection periods [e.g. by reverse transcription polymerase chain reaction (RT-PCR)] or on the potential infectivity of the virus after an exhumation are rare. In the present study, these parameters were examined in two cases with a time span of approximately 4 months between day of death and exhumation. Using SARS-CoV-2 RT-PCR on swabs of both lungs and the oropharynx detection was possible with cycle threshold (C_t_) values of about 30 despite signs of beginning decay. RT-PCR testing of perioral and perinasal swabs and swabs collected from the inside of the body bag, taken to estimate the risk of infection of those involved in the exhumation, was negative. Cell culture-based infectivity testing was negative for both, lung and oropharyngeal swabs. In one case, RT-PCR testing at the day of death of an oropharyngeal swab showed almost identical C_t_ values as postmortem testing of an oropharyngeal swab, impressively demonstrating the stability of viral RNA in the intact corpse. However, favorable climatic conditions in the grave have to be taken into account, as it was wintertime with constant low temperatures. Nevertheless, it was possible to demonstrate successful postmortem detection of SARS-CoV-2 infection following exhumation even after months in an earth grave.

## Introduction

A question frequently encountered by medicolegal experts in the context of an impending exhumation of a corpse pertains to the odds of a positive outcome of a specific detection method in a given scenario [1]. In the past, numerous studies have been conducted to elucidate relevant factors influencing the pace of the decay process in an earth grave, whereby in addition to ambient temperature (season), e.g. soil conditions and clothing of the corpse, have been examined [2, 3]. Reasons for an exhumation include, among others, charges filed for suspected homicides, cases pursuant to insurance law, and medical malpractice allegations [4, 5]. Existing data on the detection of bacteria or viruses in exhumed corpses are rather scarce, but may play a crucial role, for example concerning allegations regarding hygiene standards. Breitmeier et al. reported successful detection of Salmonella enteritidis after a postmortem interval (PMI) of 17 days and of influenza A after a PMI of 1.2 months in their exhumation study [2]. In the context of the current coronavirus disease 2019 (COVID-19) pandemic with an enormous death toll in the general population, a case scenario with the necessity to assess detection possibilities of severe acute respiratory syndrome coronavirus type 2 (SARS-CoV-2) after exhumation was expectable. In addition to detection of the virus in bodily fluids and organs, the question arises as to whether a corpse or its environment (coffin, body bag) are infectious after exhumation. SARS-CoV-2 was already detected by reverse transcription polymerase chain reaction (RT-PCR) on the body surface and the vicinity of cadavers, subsequent tests in the cell culture were negative [6]. In the present study, detection of SARS-CoV-2-RNA by RT-PCR and cell culture-based infectivity testing of the virus were examined in cases of exhumed decedents who died of COVID-19.

## Material and methods

### Exhumation

In April, two corpses of deceased persons, who supposedly died of COVID-19, were exhumed at the order of the public prosecutor. In the first case, exhumation was ordered to confirm a SARS-CoV-2 infection, which had antemortem been proven by SARS-CoV-2 RT-PCR. In the second case, the patient had been tested positive for SARS-CoV-2 prior to his demise only by an antigen rapid test. Subsequent medicolegal autopsy and the following additional examinations were ordered to confirm or reject SARS-CoV-2 infection and consecutive COVID-19 as the cause of death and to assess possible competing causes of death.

### Medicolegal autopsy, histological and toxicological examinations

Immediately after the exhumation, both corpses were transferred to the Institute of Legal Medicine, where a full medicolegal autopsy was carried out. In the course of both autopsies, swabs were collected at identical sites from both lungs, pleural effusion, oropharynx, perioral and perinasal regions, and the inside of the body bag at head level (an overview of the post mortem swabbing sites can also be found in Table 2). The latter three of the swabs were taken to assess a possible risk of infection for those involved in the handling of the body (e.g., morticians).

For the acquisition of the lung swabs, care was taken to avoid contamination during the process. The organ surface was 1 × disinfected with Octenisept® (Schülke & Mayr GmbH, Norderstedt, Germany) with a drying time of 1 min followed by incision of the visceral pleura with a sterile scalpel. For toxicological examination, femoral venous blood, cardiac blood, bile, urine (only in case 2), small tissue samples of brain, lung, liver, and kidney as well as hair were collected and analyzed using gas chromatography and high-performance liquid chromatography mass spectrometry.

For histological examinations, samples of the heart, lungs, liver, spleen, pancreas, kidneys, adrenal glands, thyroid gland as well as paratracheal lymph nodes were taken, fixed in 4.5% formalin solution, processed according to standard protocols and stained for hematoxylin and eosin. In both cases, the brain tissue was in a liquefied state, so no evidence could be obtained for histological examinations.

### SARS-CoV-2 RT-PCR

Swabs were suspended in 2 mL of phosphate-buffered saline (PBS) and incubated for 5 min suspended in 2 mL phosphate buffered saline (PBS) of which 500 µL was examined by RT-PCR. A total of 500 µL of the swab dilution was mixed with PCR lysis buffer (1:1 ratio) then transferred to barcoded tubes and subjected to rRT-PCR-analysis on the Cobas 6800 system (Roche Diagnostics International AG, Rotkreuz, Switzerland). The Cobas SARS-CoV-2 master mix was supplemented with an internal RNA control and primer–probe set targeting ORF1 and E-gene according to the manufacturer’s protocol. SARS-CoV-2 RT-PCR was performed according to the manufacturer’s recommendations. Positive results were reported as semi-quantitative C_t_ values. In addition, three quantitative comparison samples containing 10^5^, 10^6^, and 10^7^ SARS-CoV-2 (BetaCoV/Munich/ChVir984/2020) RNA copies/mL were used to generate a 3-point standard curve and to calculate viral RNA copies/mL [7]. The comparison samples were provided by INSTAND e.V. (Düsseldorf, Germany). In case 1, an RT-PCR test result of a swab collected on the day of death was available. Here, the cobas® 8800 platform had been used with the same reagents used for testing the postmortem samples.

No tests were carried out for SARS-CoV-2 variants of concern (VOC) or other viruses as these were not relevant at the time of examination.

### SARS-CoV-2 infectivity test using cell culture

Positive samples from the initial RT-PCR were used for cell culture experiments. Here 500 µL of the swab dilution was mixed with 2 mL of cell culture medium [minimal essential medium (MEM) containing 1% FCS (Sigma-Aldrich; St. Louis, MO, USA), 3% amphotericin B and 0.2% Primocin (InvivoGen; San Diego, CA, USA)]. These mixtures were immediately transferred to Caco-2 cells (human colon carcinoma cells, obtained from DSMZ, Braunschweig, Germany, no.: ACC 169) seeded in 5.5 cm^2^ culture tubes. Cells were incubated in a CO_2_-incubator at 37 °C for up to seven days and assessed microscopically every day for virus specific cytopathogenic effects (CPE) [12, 14]. After seven days or earlier in the case of cell lysis occurred as a sign of cell culture infection by the added virus, cell culture supernatants were tested for the presence and amount of viral RNA by RT-PCR using cobas® SARS-CoV-2 (Roche Diagnostics), to verify that CPE were attributable to SARS-CoV-2 infection.

## Results

### Exhumation

Both corpses had been put into closed plastic body bags and then buried in a wooden coffin (with burial depth of 120 cm, Table 1). Upon exhumation both coffin lids showed partial collapses with still undamaged body bags. The external state of preservation of the body differed in that in case 1, signs of decay as could be expected in a humid environment (in particular, skin loosening/slippage, moderate tissue softening), whereas in case 2, a distinct mold infestation of the face was found. Both corpses were vested with a nightdress. In both cases, no signs of preservation measures were found.

**Table 1:** Age and gender of the deceased, pre-existing conditions, time span between death and exhumation, and burial depth (measured from surface to the top of the coffin lid)

Case	Gender	Age/y	Pre-existing conditions	Time span death to exhumation/d	Burial depth/cm
1	f	> 90	Dementia, chronic renal insufficiency, arterial hypertension, peripheral artery disease	4 mo 0 d	120
2	f	> 80	dementia, arterial hypertension, epilepsy	3 mo 26 d	120

### Medical history

In case 1, the woman had died 4 months before exhumation. The funeral took place 8 days after her death. Her medical records stated that she had suffered from dementia, chronic renal insufficiency, arterial hypertension, and peripheral artery disease. Thirteen days prior to death, she had been tested positive using a rapid SARS-CoV-2 antigen test, after initial symptoms of fever and fatigue had been noted 15 days prior to death (Table 2). One day before her death, the woman was admitted to a hospital in a state of strong dyspnea. Upon arrival, she was resuscitated but died a few hours later. A SARS-CoV-2 RT-PCR test of an oropharyngeal swab on the date of her death showed a C_t_ value of 30.19 (ORF1 gene).

**Table 2:** Results of postmortem virological examinations and data on medical history of both cases; no cell culture was assessed in case of a negative RT-PCR result. *RT-PCR*, reverse transcription polymerase chain reaction; *CPE*, cytopathogenic effect; *C*_*t*_, cycle threshold

	Case 1			Case 2		
Time span symptom onset to death	15 d			19 d		
Time span positive rapid antigen test to death	13 d			17 d		
Antemortem RT-PCR/C_t_-value (ORF1 gene)/swab localization	30.19 (= 43.745 RNA copies/mL)/oropharynx			Not collected		
Symptoms	Fever, fatigue, deterioration of general condition			Fever, fatigue, diarrhea, flu-like symptoms		
Postmortem swab localization	RT-PCR (C_t_-value ORF1 gene)	RNA copies/mL	cell culture (CPE)	RT-PCR (C_t_-value ORF1 gene)	RNA copies/mL	cell culture (CPE)
Oropharynx	32.10	11.445	Negative	29.02	99.457	Negative
Right lung	31.42	18.447	Negative	30.17	44.364	Negative
Left lung	29.16	90.147	Negative	29.90	53.622	Negative
Pleural effusion	Negative		Not examined	Negative		Not examined
Perioral/perinasal	Negative		Not examined	Negative		Not examined
Inner surface of body bag	Negative		Not examined	Negative		Not examined

In case 2, the woman had died 3 months and 26 days before exhumation. In her medical records, dementia, arterial hypertension, and epilepsy were listed as chronic pre-existing conditions. A rapid SARS-CoV-2 antigen test conducted 17 days before the death was positive, after she had displayed fever as an initial symptom 19 days prior to death (Table 2). A PCR test was not performed during her lifetime. In this case, there was no admission to a hospital in the course of the disease. The woman was buried 5 days after her death.

### Medicolegal autopsy, histological and toxicological examinations

**Fig. 1:**
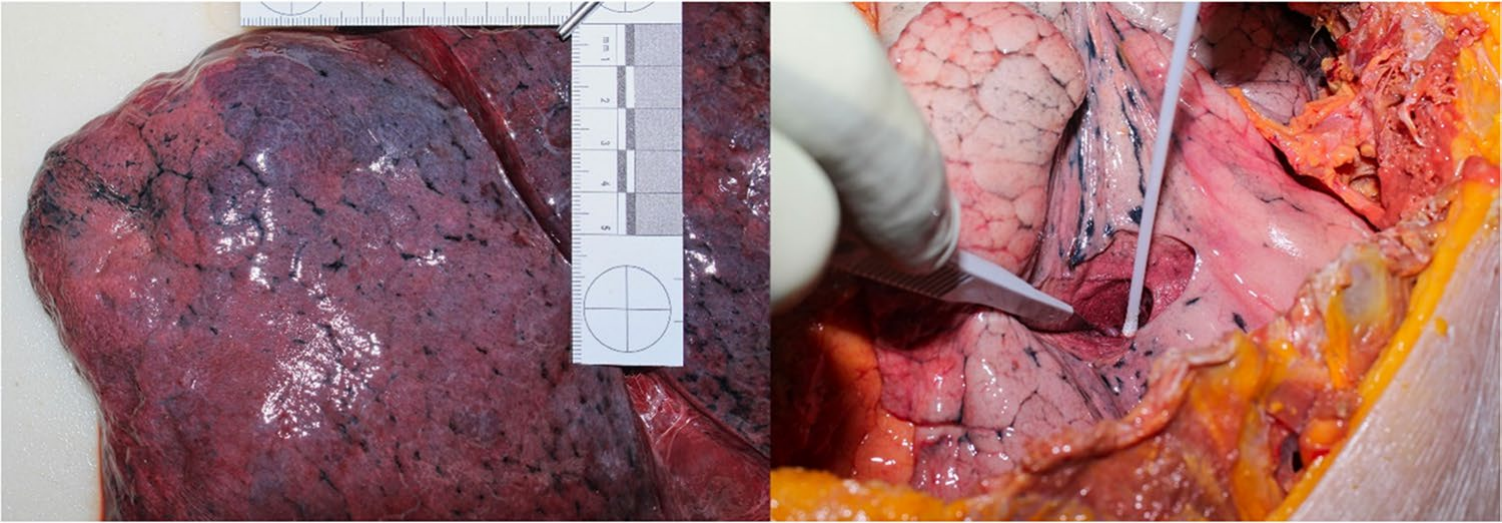
Macroscopical aspect of the lungs (left: case 1, right: case 2 — during the collection of samples for virological examinations)

Upon medicolegal autopsy, both corpses showed moderate to advanced signs of external and mild to moderate signs of internal decay. The lungs were particularly affected to a lesser degree upon macroscopical examination (Fig. 1).

In case 1, both lungs showed a condensed and solidified state as in prolonged inflammation. Furthermore, all three main branches of the coronary arteries showed coronary heart disease while a general atherosclerosis and signs of renal atherosclerosis were noted. Examination of the heart yielded a mitral valve insufficiency and aortic valve stenosis.

In case 2, the lung tissue was intensely solidified and somewhat friable in the central parts of lobes as in prolonged inflammation. The heart showed left ventricular hypertrophy (wall of the left ventricle: 2.4 cm; heart weight: 320 g), coronary heart disease of the left anterior descending and circumflex, mitral valve insufficiency, and aortic valve stenosis. Upon further inspection, general atherosclerosis and signs of renal atherosclerosis were noted.

Histologically, lung affection of decay was more pronounced than expected based on macroscopical findings. There were accumulations of bacteria, partly blurred cell boundaries, cell shadows and sometimes unstained cell nuclei in all organs of both cases.

In case 1, interstitial accumulation of lymphocytes and, as far as it could be assessed in the presence of decay, a mild hemorrhagic edema and alveolar hyaline membranes as signs of an acute respiratory distress syndrome (ARDS) in its exudative phase were present. In addition, signs of moderate chronic obstructive pulmonary disease (COPD) were seen.

In case 2, lungs displayed an intense intraalveolar edema, interstitial accumulations of lymphocytes, broadened alveolar septa, and numerous patchy accumulations of granulocytes. In addition, cardiac hypertrophy could be shown.

Postmortem toxicological analysis demonstrated the drugs fentanyl, midazolam, propofol, norephedrine and amantadine in case 1 (having gone through clinical resuscitation). Drug concentrations were subtherapeutic or therapeutic and thus did not indicate an intoxication. The blood alcohol concentration was 0.45 ‰, which may be attributed to endogenous ethanol production in the postmortem phase.

In case 2, the drugs levetiracetam, fentanyl and bisoprolol were found to be of no relevance to death in terms of their concentration. The blood alcohol concentration was 0.08 ‰ and, as in case 1, attributed to postmortem changes.

### Virological examinations

Swabs collected from the oropharynx and lungs of both cases tested positive for SARS-CoV-2 in the RT-PCR with C_t_ values ranging from 29.16 to 32.10 in case 1 and 29.02 to 30.17 in case 2, respectively, whereas RNA copies/mL ranged from 11.445 to 90.147 in case 1 and 44.364 to 99.457 in case 2 (Table 2). In case 1, 4 months after death, viral RNA copies in the oropharyngeal swab were reduced by a factor of approximately 3.8 as compared to the antemortem findings. In addition, PCR-positive samples were tested for infectivity in cell culture. None of the six samples showed a cytopathogenic effect after seven days, so there was no longer any measurable infectivity. The cell culture supernatants were subsequently tested for SARS-CoV-2-RNA by RT-PCR and all showed a positive result, which reflects the inoculated sample material.

### Cause of death

In both cases, COVID-19 pneumonia was ascertained as the cause of death. In case 2, COVID-19 pneumonia had been complicated by bacterial superinfection.

## Discussion

In the medical literature, there are hardly any publications on the detection of viruses in exhumated corpses, in particular after a moderate postmortem interval. A comparable time span has been published for the detection of HCV-RNA [8], whereas positive testing at an early point in time after burial has been shown for influenza (1.2 months [2]) and recently for SARS-CoV-2, where RT-PCR testing showed positive results in lung and heart tissue of a case one month after death with C_t_ values of 31 and 36, respectively [9]. In their study, no infectivity could be detected by the use of cell culture [9]. Prasad et al. showed that SARS-CoV-2 could be detected by RT-PCR from a nasopharyngeal swab of an exhumed corpse 36 days after death and pointed out the high stability of the virus [10].

In the present study, detection of SARS-CoV-2 by RT-PCR in lung and oropharyngeal swabs was possible in two cases after a time span of four months between death and exhumation. In addition, samples of the body surface and the environment of the body were tested to identify potential risks in the handling of the body during and after exhumation for involved personnel. Neither perioral nor perinasal skin swabs, nor those collected from the inside of the body bags, were positive in RT-PCR testing. In those samples with RT-PCR positive lung and oropharyngeal swabs, inoculated cell culture showed no cytopathogenic effect as an indicator of infectivity.

Despite these findings, a generalized assessment as non-infectious (of corpses in general or tissue and body fluid samples in particular) should not be derived from the results of our experiments. In clinical (living) patients, infectivity has been demonstrated up to 12 days after the onset of symptoms in the wild type virus [11]. In case 1 of our study, the first symptoms occurred 15 days and in case 2, 19 days before death, so it is possible that both patients were no longer infectious due to the duration of the disease at the time of death. Studies on postmortem changes in COVID-19 patients have produced evidence that moderate signs of decay in a corpse will not have a major influence on the infectivity of the virus [12]. In their study with samples of living patients, La Scola et al. showed that infectivity in cell culture correlates with the level of viral RNA load, with CPE being observed at low C_t_ values rather than at higher ones [13]. Also, Kohmer et al. were able to demonstrate that, using the same reagents and cell culture systems as in this study, only in < 15% of swab samples containing viral amounts of < 10^5^ RNA/mL infectivity was shown [14]. In our study, the RNA copy count/mL was always below 10^5^, so that a low viral RNA load can be assumed.

The postmortem detection of SARS-CoV-2 by RT-PCR was also successful in other studies after medium periods of time, for example 12 or 35 days after death [15, 16]. A very peculiar finding presented in our study is that, an RT-PCR test result from the day of death was available, which, because the same test system was used, could be directly compared with the results of the postmortem oropharyngeal swab. When comparing the quantitative values, postmortem RNA copies/mL were approximately 3.8 times lower as at the day of death. As a 3.8-fold decrease is within the normal fluctuation range of the test system used, the viral load may be assumed as rather stable in the intact body over a prolonged postmortem time.

In addition, our data show that postmortem SARS-CoV-2 RT-PCR testing can be successful not only in cases with initially low C_t_-values < 25, but also in cases, in which, at the time of death, there are moderate to lower virus quantities (with C_t_-values ~ 30 or higher). In terms of the stability of SARS-CoV-2-RNA, soil temperature is likely to play a significant role. As it was winter, soil temperature should have been rather stable at around 8° C [17], likely to contribute to the successful testing as SARS-CoV-2 is known to have higher stability at lower temperatures [18].

This study is subject to some limitations. There were only two cases available to be included in the study, as the combination of exhumation and SARS-CoV-2 positivity is rare — exhumations are rather seldomly performed in Germany (20 cases in our institute in the last 10 years) and SARS-CoV-2 with its first description in December 2019 [19] is to be regarded as a new virus. Although an advantage of the study may be seen in the fact that both cases had an almost identical time span spent in the earth grave and exhumations were performed almost contemporaneously in spring, rendering the results comparable as pertains to these circumstances, results may not be exported uncritically and employed for examinations elsewhere without regarding air and soil temperature conditions, and soil conditions in general.

## Conclusions

RT-PCR-based detection of SARS-CoV-2 in corpses of COVID-19 patients is possible even after 4 months in an earth grave, when temperature and grave conditions are comparable to those in our study. Oropharyngeal as well as lung swabs yielded positive results and in the one case, where antemortem viral quantification had been performed, the viral load was shown to be rather stable as compared to postmortem material. None of the swabs testing positive for SARS-CoV-2 proved to be infectious as demonstrated by cell culture inoculation. These results should not be overinterpreted in the sense of a general non-infectious state of exhumated corpses having died from COVID-19, as inter alia both cases included in this study had time spans between initial symptoms and death of ≥ 15 days.
